# Identification of Ras-degrading small molecules that inhibit the transformation of colorectal cancer cells independent of β-catenin signaling

**DOI:** 10.1038/s12276-018-0102-5

**Published:** 2018-06-06

**Authors:** Wookjin Shin, Sang-Kyu Lee, Jeong-Ha Hwang, Jong-Chan Park, Yong-Hee Cho, Eun Ji Ro, Yeonhwa Song, Haeng Ran Seo, Kang-Yell Choi

**Affiliations:** 1 Translational Research Center for Protein Function Control, Yonsei University, Seoul, Republic of Korea; 2 Department of Biotechnology, College of Life Science and Biotechnology, Yonsei University, Seoul, Republic of Korea; 3 Cancer Biology Research Laboratory, Institut Pasteur Korea, Seongnam-si, Gyeonggi-do Republic of Korea

**Keywords:** Cell growth, Drug development

## Abstract

Although the development of drugs that control Ras is an emerging topic in cancer therapy, no clinically applicable drug is currently available. We have previously utilized knowledge of the Wnt/β-catenin signaling-dependent mechanism of Ras protein stability regulation to identify small molecules that inhibit the proliferation and transformation of various colorectal cancer (CRC) cells via degradation of both β-catenin and Ras. Due to the absence of Ras degradation in cells expressing a nondegradable mutant form of β-catenin and the need to determine an alternative mechanism of Ras degradation, we designed a cell-based system to screen compounds that degrade Ras independent of the Wnt/β-catenin signaling pathway. A cell-based high-content screening (HCS) system that monitors the levels of EGFP-K-Ras^G12V^ was established using HCT-116 cells harboring a nondegradable mutant *CTNNB1* (ΔS45). Through HCS of a chemical library composed of 10,000 compounds and subsequent characterization of hits, we identified several compounds that degrade Ras without affecting the β-catenin levels. KY7749, one of the most effective compounds, inhibited the proliferation and transformation of CRC cells, especially KRAS-mutant cells that are resistant to the EGFR monoclonal antibody cetuximab. Small molecules that degrade Ras independent of β-catenin may able to be used in treatments for cancers caused by aberrant EGFR and Ras.

## Colorectal cancer: Finding new small molecule drug candidates

Mutations in *KRAS*, a gene regulating cell proliferation, occur in 40–50% of colorectal cancer (CRC) patients. These cancers are usually insensitive to antibody drugs targeting the epidermal growth factor receptor (EGFR) on cancer cells. Kang-Yell Choi at Yonsei University, Seoul, South Korea and co-workers had previously identified small molecules that inhibited CRC by degrading the signaling proteins β-catenin and Ras. However, CRC cells with β-catenin mutations are resistant to Ras-degrading compounds. In this study, they screened 10,000 small molecules that could degrade Ras in CRC cells expressing nondegradable mutant β-catenin. They identified small molecules that inhibited growth of CRC cells resistant to EGFR-mediated antibody therapy. Further investigation of these molecules may help develop new drugs for treating CRC.

## Introduction

Mutations in the Ras family of small GTPase genes, especially *KRAS*, frequently occur in human cancers, including colorectal cancer (CRC), and are associated with poor prognosis and poor response to standard cancer therapies^1–3^. The treatment of cancers caused by *Ras* mutations, especially *KRAS* mutations, is an important issue to overcome as patients harboring *KRAS* mutations are insensitive to the current target-specific anticancer drugs cetuximab and panitumumab, both of which are epidermal growth factor receptor (EGFR) antibodies^4–6^. Since *Ras* mutations were identified in human cancers more than 35 years ago, numerous studies have identified anticancer drugs that control oncogenic Ras activity^7, 8^. However, efforts to control oncogenic Ras activity using small molecules that directly interact with Ras, indirect approaches to inhibit the membrane localization of Ras using farnesyltransferase inhibitors^9, 10^, and targeting downstream effectors^11–14^ have not been successful. Several alternative approaches to control Ras, such as siRNA-mediated knockdown combined with nanotechnology^15, 16^ and K-Ras (G12C) inhibitors that allosterically control GTP affinity and effector interactions^17–19^ are being developed. However, a clinically applicable drug that controls oncogenic Ras is not available.

As an alternative to develop anticancer drugs targeting Ras, we recently identified and characterized small molecules that reduce Ras activity by inducing the degradation of Ras and β-catenin through the Wnt/β-catenin pathway^20, 21^. KYA1797K, a small molecule, efficiently inhibited the proliferation and transformation of various CRC cells expressing high levels of β-catenin and Ras as a result of *APC* loss, which occurs in up to 90% of human CRC patients^20^. KYA1797K and its analogs are especially effective on CRC cells harboring both *APC* and *KRAS* mutations; these mutations synergistically promote tumorigenesis through the stabilization of oncogenic K-Ras and β-catenin^22^. KYA1797K induced Ras degradation via direct binding at the RGS domain of Axin, stimulating *GSK3β*-mediated phosphorylation of Ras at threonine-144 (Thr-144) and Thr-148^20^. However, Ras degradation did not occur in KYA1797K-treated CRC cells expressing a nondegradable mutant form of *CTNNB1* (which encodes β-catenin)^20^. In addition, we observed that a subpopulation of Ras protein was degraded independent of *GSK3β*, the key enzyme responsible for the phosphorylation-dependent degradation of both β-catenin and Ras^23^. These results indicated the presence of an alternative Wnt/β-catenin-independent mechanism controlling Ras degradation.

Therefore, to identify small molecules that promote Ras degradation independent of Wnt/β-catenin signaling involving *GSK3β*, we established a high-content screening (HCS) system using the *CTNNB1* mutant HCT-116 CRC cell line. To sensitively and efficiently detect changes in Ras levels, especially the oncogenic mutant form, we generated HCT-116 cells that stably expressed EGFP-K-Ras^G12V^. Small molecules that reduced the green fluorescent protein (GFP) signal in these cells were identified by screening a chemical library composed of 10,000 compounds. Several small molecules were identified, and KY7749, a compound that significantly reduced Ras levels without reducing β-catenin levels, was selected for further characterization based on fluorescence-activated cell sorting (FACS) and immunoblotting analyses. The ability of KY7749 to inhibit the proliferation of CRC cells harboring *KRAS* and *CTNNB1* mutations correlated with its ability to promote Ras degradation. Moreover, the degradation of mutant K-Ras following KY7749 treatment resulted in the inhibition of proliferation, transformation, and migration of *KRAS*-mutant CRC cells. Therefore, this drug could be used to circumvent cetuximab resistance in CRC patients harboring *KRAS* mutations. KY7749 and its analogs could be useful as anticancer drug candidates for the treatment of CRC patients and other types of cancer patients who harbor *RAS* mutations or overexpress EGFR.

## Materials and methods

### Cell lines, culture conditions, and reagents

HCT-116 and SW48 CRC cells were obtained from the American Type Culture Collection (ATCC, Manassas, VA, USA). SW480 and DLD-1 CRC, HEK293, and the mouse embryonic fibroblast (MEF) *GSK3β*^+/+^ and *GSK3β*^−/−^ cell lines were described in a previous study^20^. Isogenic human DLD-1 CRC cells expressing either wild type (WT) or mutant (MT) *KRAS* (D-WT and D-MT cells, respectively) were described in a previous study^22^. HCT-116 and SW48 cells were maintained in RPMI 1640 medium (Gibco Life Technologies, Grand Island, NY, USA) containing 10% heat-inactivated fetal bovine serum (Gibco Life Technologies) and 1% penicillin–streptomycin (Gibco Life Technologies). Cycloheximide (CHX; 50 µg ml^−1^; R&D Systems, Minneapolis, MN, USA) and the proteasome inhibitor MG132 (20 μM; Calbiochem) were added to the media to inhibit protein synthesis and proteasomal degradation, respectively. Radioimmunoprecipitation assay (RIPA) buffer (Upstate Biotechnology, Lake Placid, NY, USA) was used for cell lysis. N-ethylmaleimide (Sigma-Aldrich, St. Louis, MO, USA) was added to the RIPA buffer for ubiquitination assays. Cetuximab (Erbitux^®^) was provided by Merck KGaA (Darmstadt, Germany). All chemicals were dissolved in dimethyl sulfoxide (DMSO; Sigma-Aldrich) for in vitro studies.

### Plasmid construction and generation of stable cell lines

Human K-Ras, H-Ras, and N-Ras cDNA fragments were cloned into the pcDNA3.1-Myc vector in a previous study^23^. The substitution of human K-Ras threonines 144 and 148 to alanines (T144/148 A) was described in a previous study^20^. The pFA2-Elk-1, pFR-Luc, and pCMV-β-gal reporter plasmids were used for Elk-1 reporter assays^23^. The pEGFP-C3 and pEGFP-K-Ras^G12V^ plasmids were provided by Dr. Yoel Kloog (Tel-Aviv University, Tel-Aviv, Israel). EGFP and EGFP-K-Ras^G12V^ cDNA fragments were cloned into the pLVX-IRES-Hygro (Clontech, Mountain View, CA, USA) vector using the *XBa*Ι/*Not*Ι and *XBa*Ι/*Bam*HΙ sites, respectively. The lentiviral packaging and virus production procedures were described in a previous study^24^. To generate HCT-116 cells stably expressing EGFP or EGPF-K-Ras^G12V^, HCT-116 cells were infected with lentivirus-containing media with polybrene (8 µg ml^−1^) for 6 h. All cells were selected using hygromycin (50 µg ml^−1^; Sigma-Aldrich) for 35 days.

### High-content drug screening

A chemical library set containing 10,000 compounds (ChemDiv, San Diego, CA, USA) was used for HCS. HCS was performed in clear-bottom 384-well plates (Greiner Bio-One, Monroe, NC, USA). Parental HCT-116 cells and HCT-116 EGFP-K-Ras^G12V^ cells were seeded at a density of 8 × 10^3^ cells per well in 384-well plates for 16 h. Parental HCT-116 cells were used as a baseline positive control. Each chemical compound was added individually to the HCT-116 EGFP-K-Ras^G12V^ cells at a final concentration of 10 μM using a JANUS Automated Workstation (PerkinElmer, Shelton, CT, USA). After a 24 h incubation, the plates were washed with Dulbecco’s Phosphate-Buffered Saline (DPBS; Gibco) using a Microplate Dispenser (Thermo, San Diego, CA, USA). Cells were fixed in 4% paraformaldehyde (PFA; Wako, Richmond, VA, USA) and stained with Hoechst33342 (Invitrogen, Waltham, MA, USA). All plates were scanned and analyzed using the Operetta High-content Imaging System (PerkinElmer).

### Immunoblotting, immunoprecipitation, and ubiquitination assays

Immunoblotting was performed as previously described^25^ using the following primary antibodies: anti-pan-Ras, anti-K-Ras, anti-H-Ras, anti-N-Ras, anti-β-catenin, anti-ERK, anti-p-ERK, anti-PCNA, anti-Myc, anti-GFP, and anti-α-tubulin antibodies as described in a previous study^20^. Immunoprecipitation and ubiquitination assays were performed as previously described^25^ using anti-pan-Ras, anti-β-catenin, or anti-α-tubulin antibodies as described in a previous study^23^. Horseradish peroxidase-conjugated anti-mouse or anti-rabbit secondary antibodies were used as described in a previous study^20^.

### Ras GTP loading assay

The Ras GTP loading assay was performed as described previously^24, 26^. In brief, cell lysates were incubated with a bacterially produced glutathione S-transferase fusion protein corresponding to the Ras-binding domain of Raf-1 (GST-RBD) conjugated to agarose beads. Protein-bound beads were washed three times with lysis buffer (10% glycerol, 50 mM Tris-HCl pH 7.4, 100 mM NaCl, 1% NP-40, 2 mM MgCl_2_), eluted in 1 × sodium dodecyl sulfate (SDS; AMRESCO, Solon, OH, USA) sample buffer, and analyzed for GTP-Ras by immunoblotting with anti-pan-Ras and anti-GST antibodies.

### Cell proliferation and colony formation assays

For the cell proliferation assays, HCT-116, SW48, DLD-1, and SW480 cells were seeded at a density of 5 × 10^3^ cells per well in 96-well plates for 16 h, and then cultured with KY7749, KYA1797K, or cetuximab for 0, 24, 48, 72, and 96 h. Next, 0.25 mg ml^−1^ 3-(4,5-dimethylthiazol-2-yl)-2-5-diphenyltetrazolium bromide (MTT; AMRESCO) reagent was added to each well. After a 2 h incubation at 37 °C, insoluble purple formazan was obtained by removing the medium and incubating cells in 100 μl of DMSO for 1 h. The absorbance of the formazan product was determined using a FLUOstar^®^ OPTIMA (BMG Labtech, Cary, NC, USA) at 590 nm. For colony formation assays, HCT-116, SW480, D-WT, and D-MT cells were seeded at a density of 8 × 10^2^ to 1 × 10^3^ cells per well in 12-well plates for 16 h and then cultured with KY7749 or cetuximab. The medium was changed every three days until visible colonies formed. At the end of the experiment, the cells were fixed in 4% PFA for 30 m and stained with 0.5% Crystal Violet (Sigma-Aldrich) in 20% ethanol (Sigma-Aldrich) for 30 min. All experiments were performed in at least three independent replicates.

### Migration assay

LoVo cells were seeded at a density of 3.5 × 10^5^ cells per well in 12-well plates. After reaching 100% confluence, the cell monolayer was scratched with a 200 μl pipette tip and washed with DPBS. Wounded cells were cultured with KY7749 or cetuximab for 18 h, fixed in 4% PFA for 30 min, and stained with 0.5% Crystal Violet in 20% ethanol for 30 min. The area containing migrated cells was analyzed and quantified using NIS-Elements AR 3.1 software (Nikon, Melville, NY, USA). All experiments were performed in at least three independent replicates.

### Flow cytometry analyses

HCT-116, HCT-116 EGFP, and HCT-116 EGFP-K-Ras^G12V^ cells were seeded at a density of 3 × 10^5^ cells per well in 6-well plates for 16 h and then treated with KY2776, KY0971, KY7749, or KY1311 for 24 h. Following treatment, the cells were harvested and resuspended in binding buffer (10 mM HEPES, 140 mM NaCl, and 2.5 mM CaCl_2_) at 1 × 10^6^ cells per ml. Fluorescence was analyzed on a BD Accuri C6 (BD Bioscience, San Jose, CA, USA), and the median signal for each sample was normalized to controls. To avoid interference effects by the compounds themselves, the values obtained from each compound were compensated using values from the drug-treated HCT-116 parental cells. All experiments were performed in at least three independent replicates.

### Reverse transcription and polymerase chain reaction (PCR)

HCT-116 cells were seeded at a density of 3 × 10^5^ cells per well in 6-well plates for 16 h and then treated with KY7749. After a 24 h incubation, the cells were harvested and total RNA was isolated as previously described^20^. For cDNA synthesis, 4 µg of RNA was reverse transcribed using 200 units of reverse transcriptase (Invitrogen) in a 40 μl reaction carried out at 42 °C for 1 h. For reverse transcription PCR analyses, 1 μl of cDNA was amplified using AccuPower® PCR PreMix (Bioneer, Daejeon, Republic of Korea) and 10 pM of the primer set (Bioneer) in a 40 μl reaction; *GAPDH* served as a loading control. For quantitative real-time PCR analyses, 1 μl of cDNA was amplified using iQ^™^ SYBR^®^ Green Supermix (Bio-Rad, Hercules, CA, USA) in a 10 μl reaction. The comparative cycle-threshold (C_T_) method was used, and *ACTB* served as an internal control. The following primer sets were used: *KRAS*, forward 5′-AAACAGGCTCAGGACTTAG-3′ and reverse 5′-GTATAGAAGGCATCATCAAC-3′; *HRAS*, forward 5′-GGAAGCAGGTGGTCATTG-3′ and reverse 5′-AGACTTGGTGTTGTTGATGG-3′; *NRAS*, forward 5′-AAGAGTTACGGGATTCCATTC-3′ and reverse 5′-CCATCATCACTGCTGTTGA-3′; *GAPDH*, forward 5′-ATTGTCAGCAATGCATCCTG-3′ and reverse 5′-GTAGGCCATGAGGTCCACCA-3′; *CTNNB1*, forward 5′-ACAAGCCACAAGATTACAAGAA-3′ and reverse 5′-GCACCAATATCAAGTCCAAGA-3′; and *ACTB*, forward 5′-CTGGTAAAGTGGATATTGTTG-3′ and reverse 5′-TGGAAGATGTGATGGGATTT-3′. All experiments were performed in at least three independent replicates.

### Statistical Analyses

All statistical analyses and calculations were performed as previously described^22^. Statistical significance was assessed by two-sided student’s *t* test (*n.s*., not significant; ^*^p < 0.05; ^**^p < 0.01; ^***^p < 0.005).

## Results

### Identification of small molecules that degrade oncogenic K-Ras independent of β-catenin

**Fig. 1:**
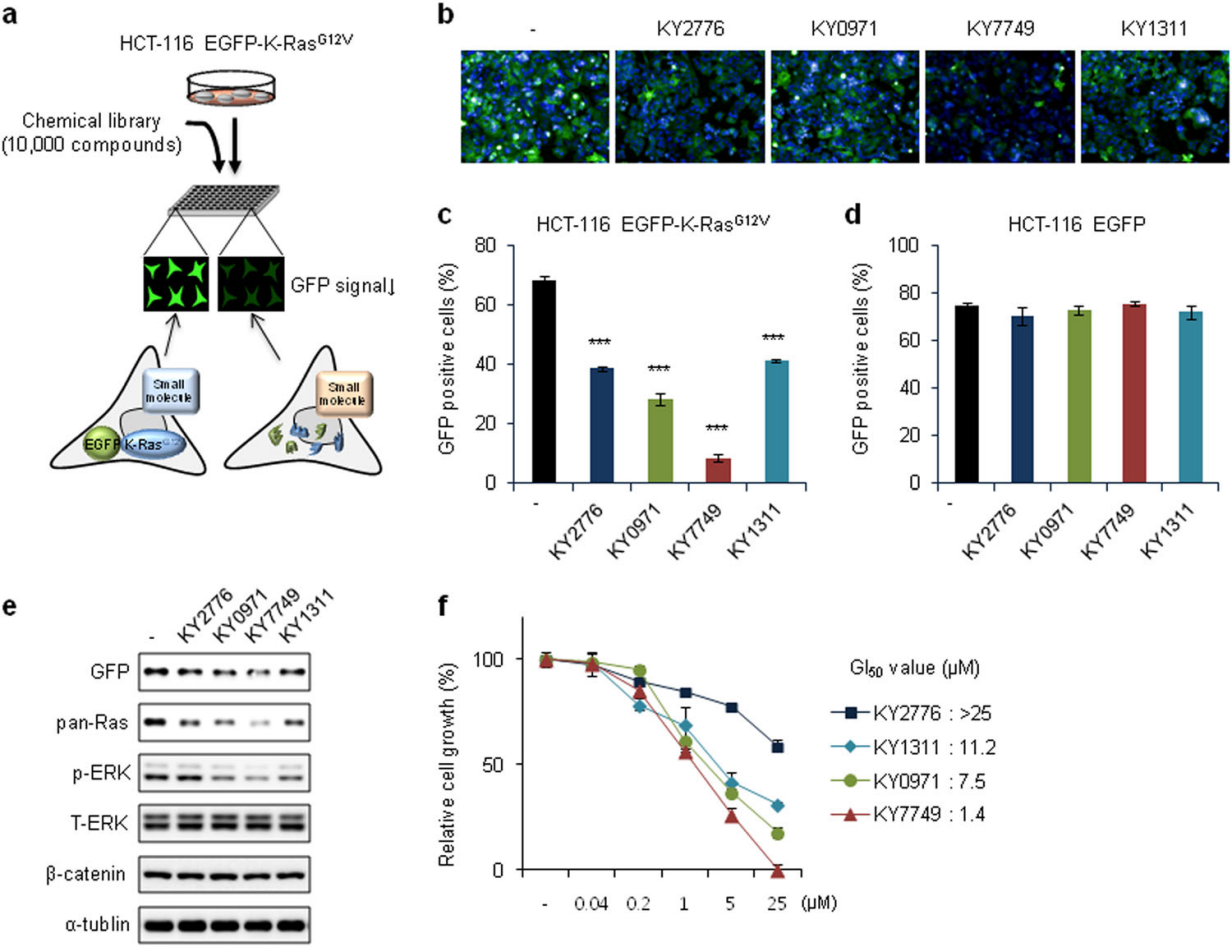
High-content screening (HCS) and characterization of small molecules that degrade oncogenic K-Ras in CRC cells with *CTNNB1* mutations. **a** A schematic of the method used to identify small molecules that degrade mutant K-Ras (G12V) in HCT-116 cells expressing nondegradable mutant β-catenin. The established HCT-116 EGFP-K-Ras^G12V^ cells were cultured in 384-well plates, and small molecules that reduced the green fluorescent protein (GFP) signal were selected as initial positive hits, as described in the results. **b** Fluorescent images from four representative hit compounds selected by HCS. **c**, **d** Flow cytometry analysis of the four hit compounds based on fluorescence of HCT-116 EGFP-K-Ras^G12V^ (**c**) or HCT-116 EGFP (**d**) cells treated with 10 μM of each compound for 24 h (mean ± SD, *n* = 3). **e** Immunoblot analyses of HCT-116 EGFP-K-Ras^G12V^ cells treated with 10 μM each of the four hit compounds for 24 h. **f** MTT [3-(4,5-dimethylthiazol-2-yl)-2,5-diphenyltetrazolium bromide] assays in HCT-116 EGFP-K-Ras^G12V^ cells treated with different concentrations of each hit compound for 96 h (mean ± SD, *n* = 3). Relative cell proliferation was normalized to dimethyl sulfoxide (DMSO)-treated controls and 50% growth inhibition (GI_50_) was calculated. Whole cell lysates (WCLs) from each of the cells were immunoblotted with antibodies against the indicated proteins (**e**)

To screen small molecules that promote the degradation of K-Ras protein, especially the mutant form, we established a stable HCT-116 cell line expressing EGFP-K-Ras^G12V^ using lentivirus-mediated chromosomal gene incorporation. The robustness of our HCS system was validated using the Z′ factor (0.43), which was calculated based on the positive control, the parental HCT-116 cell line^27^ (Supplementary Figure S1a). To identify potential small molecule candidates, we screened 10,000 compounds using a cell-based HCS system (Fig. 1a). The change in GFP signal in cultured HCT-116 EGFP-K-Ras^G12V^ cells after compound treatment was monitored in 384-well plates (Fig. 1a and Supplementary Figure S1b, c). Among the 16 initial hit compounds (~0.2% of the total chemical library) (Supplementary Figure S1d), four compounds (KY2776, KY0971, KY7749, and KY1311) reduced the number of GFP-positive cells by more than 85% at 10 μM and were selected for further study (Supplementary Figure S1e and Fig. 1b). The cytotoxicity of these compounds was further tested using highly fragile primary neural stem cells (NSCs), which were freshly isolated from E14.5 rat embryos^20, 28^. The four compounds that did not show significant cytotoxicity were selected for further characterization (Supplementary Figure S1f). The effectiveness of the four compounds, as assessed by GFP signal reduction, was further confirmed by FACS analyses using the HCT-116 EGFP-K-Ras^G12V^ cells and HCT-116 EGFP cells to subtract for potential artifacts (Fig. 1c, d). The ability of these drugs to degrade K-Ras^G12V^ was also confirmed by immunoblotting, and KY7749 was identified as the most effective compound (Fig. 1e). KY7749 was also the most effective at inducing Ras degradation in the HCT-116, SW480, and LoVo CRC cell lines (Supplementary Figure S1g). Of the four compounds, KY7749 was also the most effective inhibitor of HCT-116 EGFP-K-Ras^G12V^ cell proliferation (Fig. 1f). The GI_50_ values of KY2776, KY0971, KY7749, and KY1311 were >25 μM, 7.5 μM, 1.4 μM, and 11.2 μM, respectively (Fig. 1f). Therefore, we selected KY7749 for further characterization.

### KY7749 degrades Ras via polyubiquitination-mediated proteasomal degradation independent of Wnt/β-catenin signaling

**Fig. 2:**
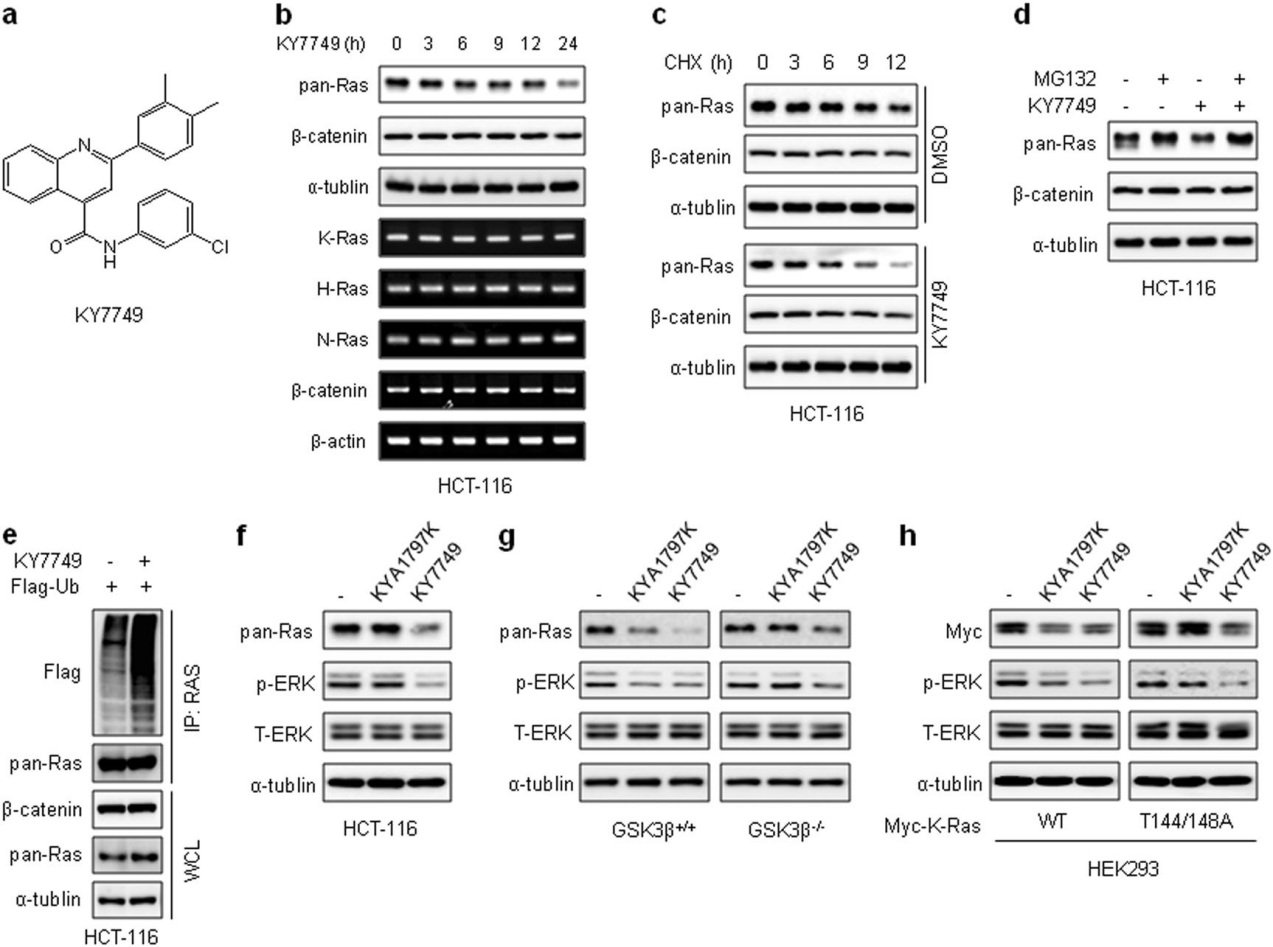
The effects of KY7749 on polyubiquitination-dependent proteasomal degradation of pan-Ras. **a** The chemical structure of KY7749. **b** Immunoblot and reverse transcription polymerase chain reaction (RT-PCR) analyses of HCT-116 cells treated with 25 μM of KY7749 for the indicated time periods. **c**Immunoblot analyses of HCT-116 cells co-treated with 50 µg ml^−1^of cycloheximide (CHX) and 25 μM of KY7749 for the indicated time periods. **d**Immunoblot analyses of HCT-116 cells treated with 25 μM of KY7749 with or without 20 μM of the proteasomal inhibitor MG132 for 4 h. **e** Immunoblot analysis of polyubiquitination-mediated proteasomal degradation of pan-Ras in HCT-116 cells transfected with Flag-Ubiquitin and then treated with 20 μM of MG132 with or without 25 μM of KY7749 for 24 h. **f**Immunoblot analysis of HCT-116 cells treated with 25 μM of KY7749 or KYA1797K for 24 h. **g**, **h** Immunoblot analysis of MEF *GSK3β*^*+/+*^ or *GSK3β* ^−*/*−^ cells (**g**) and HEK293 cells transiently overexpressing K-Ras^WT^ or K-Ras^T144/148A^ (**h**) and treated with 25 μM of KY7749 or KYA1797K for 24 h. WCLs or immunoprecipitated samples were immunoblotted with antibodies against the indicated proteins (**b**–**h**)

KY7749 consists of a 4-quinolinecarboxamide core, substituted with a 3, 4-dimethylphenyl group at position 2 of quinoline and a 3-chlorophenyl group at the carboxamide group (Fig. 2a). Normal cells, such as HEK293 and NSCs, did not show any significant toxicity after treatment with 25 μM KY7749 for up to 48 h (Supplementary Figure S2). Treatment of *CTNNB1*-mutant HCT-116 cells with KY7749 effectively reduced pan-Ras without significant changes to β-catenin (Fig. 2b). The reduction in Ras protein levels was not caused by a change in mRNA levels of any of the Ras isotypes (Fig. 2b and Supplementary Figure S3). The half-life of pan-Ras was monitored after treatment with an inhibitor of de novo protein synthesis, cycloheximide (CHX). Pan-Ras levels were decreased from 12 to 6 h following treatment of HCT-116 cells with KY7749 (Fig. 2c). In addition, KY7749-induced Ras destabilization was blocked by treatment with a proteasome inhibitor, MG132 (Fig. 2d). KY7749-induced Ras degradation occurred through a polyubiquitination-dependent proteasome degradation mechanism, as shown by the increase in ubiquitination levels following treatment with KY7749 (Fig. 2e). To confirm whether KY7749 degraded Ras via a β-catenin-independent pathway, we compared the Ras degradation effects of KY7749 with those of KYA1797K, which degrades Ras only in cells expressing WT *CTNNB1*^20^. The growth of HCT-116 cells harboring *KRAS* and *CTNNB1* mutations was effectively inhibited by treatment with KY7749, whereas growth was only marginally reduced by KYA1797K treatment (Supplementary Figure S4). Consistent with these results, the levels of both Ras and p-ERK were effectively reduced by treatment with KY7749, but not KYA1797K (Fig. 2f). The Wnt/β-catenin pathway dependency of the KY7749 effects was tested using *GSK3β*^+/+^ and *GSK3β*^−*/*−^ MEF cells^23^. KY7749 degraded Ras regardless of the presence of *GSK3β* (Fig. 2g). In addition, KY7749 had similar effects on the phosphorylation-defective K-Ras mutants (T144/T148A), which were not affected by KYA1797K due to mutations in the *GSK3β* phosphorylation sites Thr-144 and Thr-148^20, 23^ (Fig. 2h). Overall, these results suggest that KY7749 treatment promotes the degradation of Ras via a polyubiquitination-mediated proteasomal degradation pathway that is independent of Wnt/β-catenin signaling.

### KY7749 degrades Ras isotypes and inhibits their downstream signaling

**Fig. 3:**
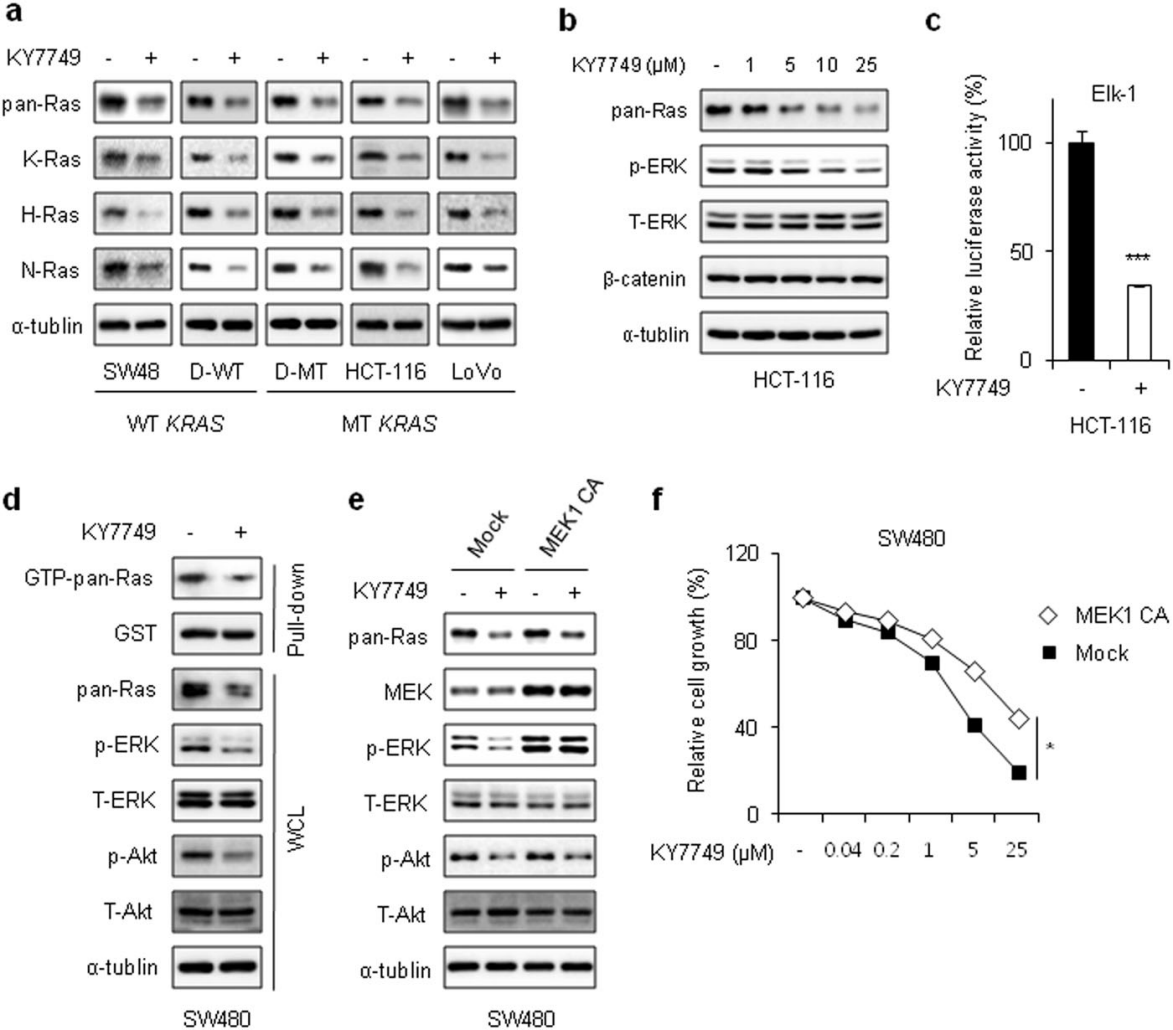
The effects of KY7749 on Ras degradation and its dependence on Wnt/β-catenin signaling. **a** Immunoblot analysis of SW48, D-WT, D-MT, HCT-116, and LoVo cells treated with 25 μM of KY7749 for 24 h. **b** Immunoblot analysis of HCT-116 cells treated with various doses of KY7749 for 24 h. **c** Elk-1 reporter assay in HCT-116 cells treated with 25 μM KY7749 for 24 h. The effect of KY7749 on ERK activity was detected based on the activity of an Elk-1 reporter construct. **d** SW480 cells were treated with 25 μM KY7749 for 24 h. GTP-bound Ras was measured as described in the Materials and Methods by pull-down of GTP-bound Ras and subsequent monitoring of phosphorylation of the Raf-1 Ras-binding domain (GST-RBD). **e** Immunoblot analysis of SW480 CRC cells overexpressing the *MEK1*CA construct and treated with 25 μM KY7749 for 24 h. **f** MTT assays in MEK1 CA-overexpressing SW480 CRC cells cultured with DMSO (control) or various doses of KY7749 for 96 h (mean ± SD, *n* = 3). Relative cell proliferation was normalized to DMSO-treated controls. WCLs or pull-down samples were immunoblotted with antibodies against the indicated proteins (**a**–**b**, **d**–**e**)

KY7749 degraded all the major K-, H-, and N-Ras isotypes in SW48, D-WT, D-MT, HCT-116, and LoVo cells regardless of their *CTNNB1* and/or *KRAS* mutational status (Fig. 3a). Consistent with these data, all K-, H-, and N-Ras proteins overexpressed in HEK293 cells were degraded by KY7749 (Supplementary Figure S5). The KY7749-induced Ras degradation effect on ERK pathway activation was confirmed by the dose-dependent inactivation of ERK and by the subsequent inhibition of the downstream Elk-1 reporter in HCT-116 cells (Fig. 3b, c). KY7749 also reduced pan-Ras levels and downstream ERK activities and inhibited D-WT cell proliferation (Supplementary Figure S6). The effect of KY7749-induced Ras destabilization on the reduction of Ras activity was confirmed by monitoring GTP-bound Ras and the inhibition of downstream ERK and Akt activities (Fig. 3d). The effect of KY7749 on ERK inactivation was demonstrated by rescue of the compound-induced ERK inactivation with overexpression of a constitutively active form (CA) of MEK1 (Fig. 3e). The inhibitory effect of KY7749 on cell proliferation was partially rescued in SW480 cells through the overexpression of MEK1 CA (Fig. 3f). The inhibitory effect of the compound could be functioning through the PI3K-Akt pathway, as shown by inhibition of Akt activity by KY7749 in cells overexpressing MEK1 CA (Fig. 3e).

### KY7749 inhibits the proliferation of CRC cells regardless of their *CTNNB1* and *KRAS* mutational status

**Fig. 4:**
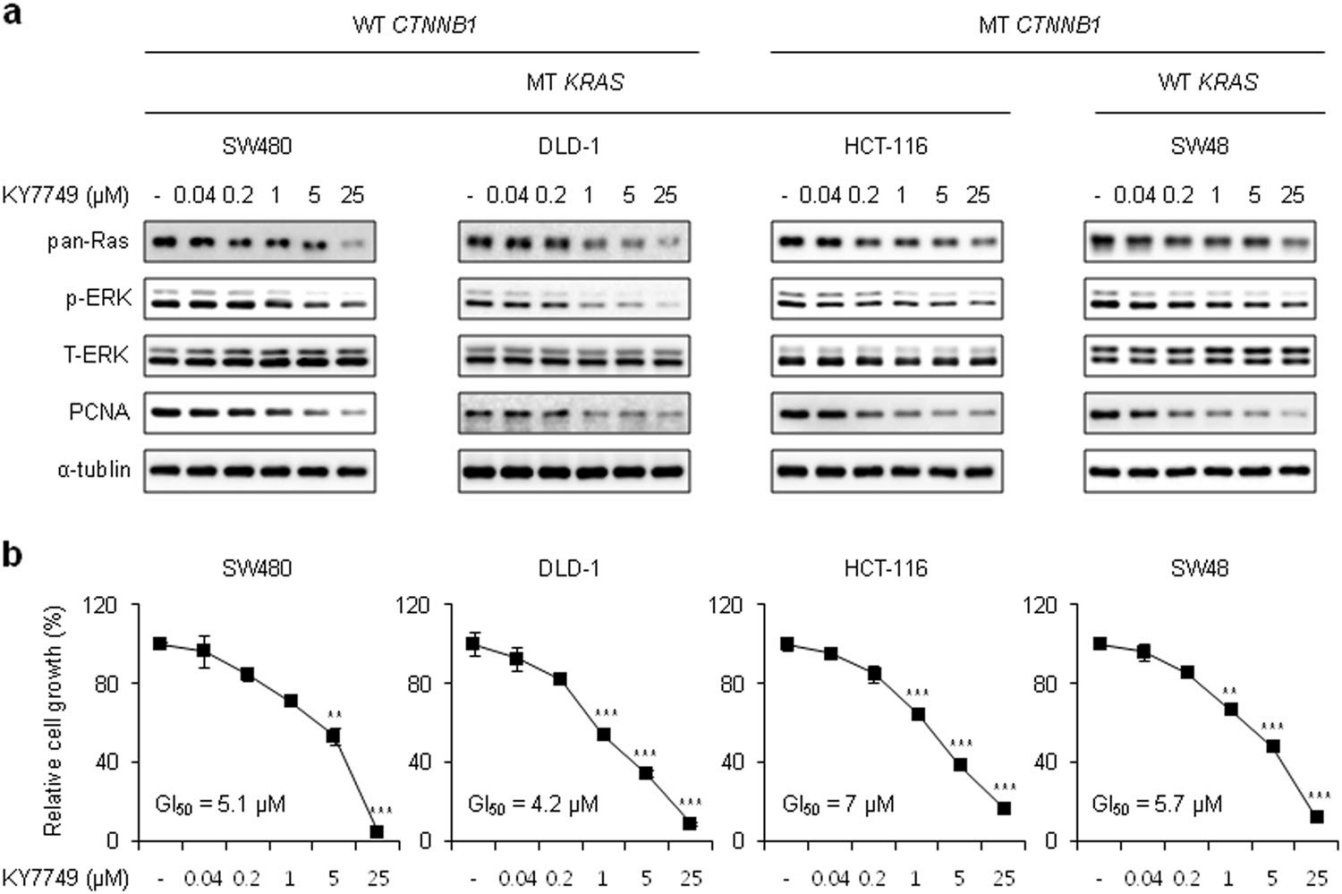
The effects of KY7749 on proliferation and levels of pan-Ras and p-ERK in CRC cells harboring WT and MT *CTNNB1* and *KRAS*. **a** Immunoblot analysis of SW480, DLD-1, HCT-116, and SW48 CRC cells treated with various doses of KY7749 for 24 h. WCLs were immunoblotted with antibodies against the indicated proteins. **b** MTT assays in SW480, DLD-1, HCT-116, and SW48 CRC cells cultured with various doses of KY7749 for 96 h (mean ± SD, *n* = 3). Relative cell proliferation was normalized to DMSO-treated controls and 50% growth inhibition (GI_50_) was calculated

To determine whether KY7749 can inhibit proliferation via destabilization of Ras in various CRC cell lines harboring *CTNNB1* and/or *KRAS* mutations, we performed immunoblotting and cell proliferation assays. KY7749 degraded Ras in SW48, HCT-116, DLD-1, and SW480 cells in a dose-dependent manner (Fig. 4a). Consistent with these results, KY7749 inhibited the growth of SW48, HCT-116, DLD-1, and SW480 cells with GI_50_ values of 7, 5.7, 4.2, and 5.1 μM, respectively (Fig. 4b). The growth inhibition patterns were correlated with reductions in proliferating cell nuclear antigen (*PCNA*) and ERK levels (Fig. 4b). These data indicate that KY7749 inhibits the proliferation of CRC cells independent of the *KRAS* and *CTNNB1* mutational status via destabilization of Ras and subsequent inhibition of ERK signaling.

### KY7749 overcomes cetuximab resistance in *KRAS*-mutant CRC cells

**Fig. 5:**
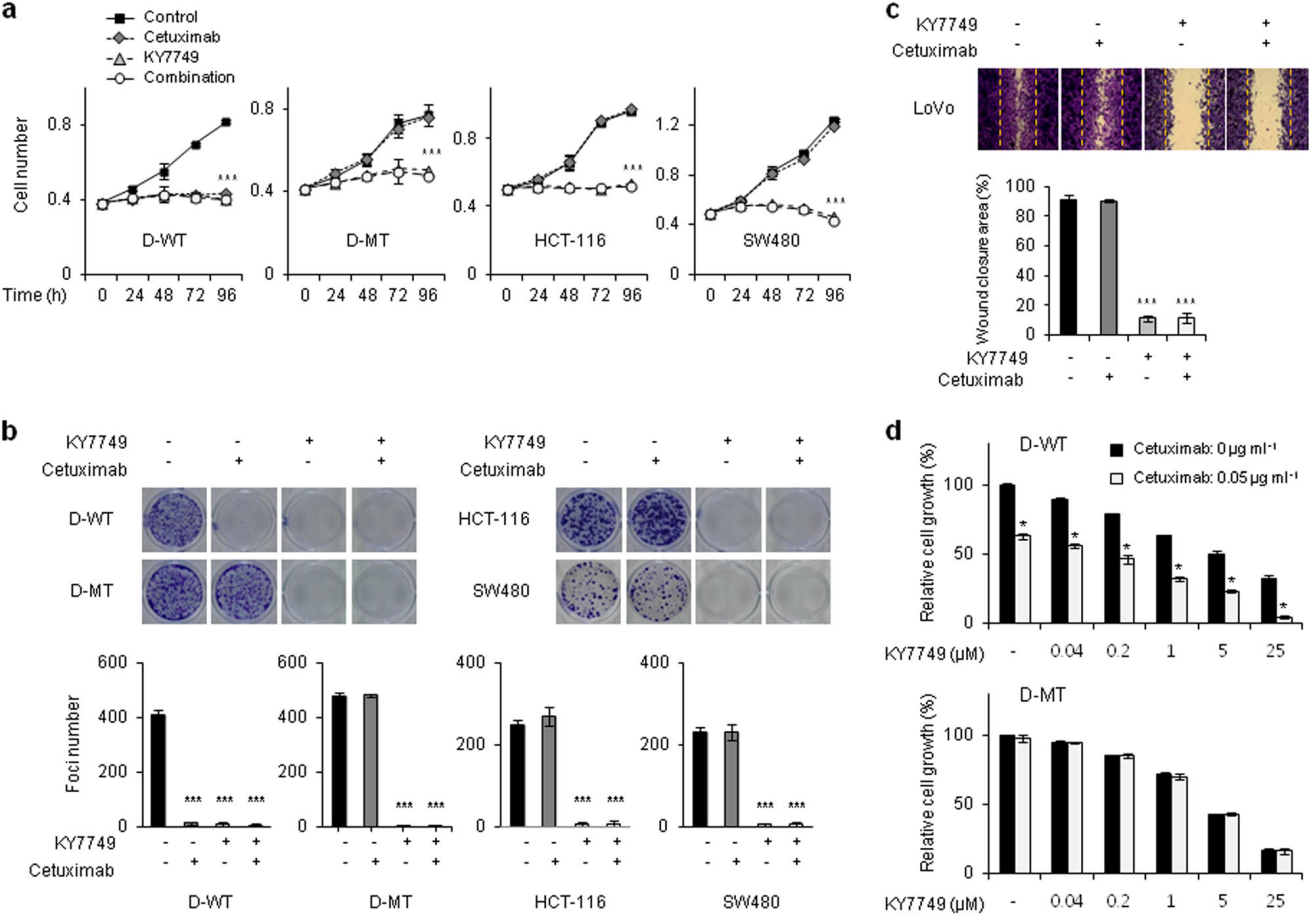
The effects of KY7749 or cetuximab on growth, transformation, and migration of CRC cells. **a** MTT assays in D-WT, D-MT, HCT-116, and SW480 cells cultured with DMSO, 25 μM KY7749, 5 μg ml^−1^ cetuximab, or KY7749 and cetuximab together for the indicated time periods (mean ± SD, *n* = 3). **b** Colony formation assays in D-WT, D-MT, HCT-116, and SW480 cells cultured with 25 μM KY7749, 5 μg ml^−1^ cetuximab, or KY7749 and cetuximab together for 13–18 days (mean ± SD, *n* = 3). **c** Migration assays of LoVo cells cultured with 25 μM KY7749, 5 μg ml^−1^ cetuximab, or KY7749 and cetuximab together for 18 h (mean ± SD, *n* = 3). Images were captured by bright field microscopy at 18 h (upper panel) to quantify the wound closure area (lower panel). **d** MTT assays in D-WT and D-MT cells co-treated with the indicated doses (0.04 to 25 μM) of KY7749 and 0.05 μg ml^−1^ cetuximab for 96 h (mean ± SD, *n* = 3)

Because the oncogenic mutant K-Ras was degraded by KY7749 treatment, we determined whether KY7749 suppressed the growth of CRC cells that were resistant to EGFR antibody therapy due to a *KRAS* mutation. The EGFR monoclonal antibody cetuximab did not inhibit the growth of D-MT, HCT-116, and SW480 CRC cells harboring *KRAS* mutations. However, cetuximab treatment did reduce the growth of D-WT cells, DLD-1 cells that uniquely harbor WT *KRAS* (Fig. 5a). Similarly, KY7749, but not cetuximab, showed anti-transformation effects but not combinatorial effects in various CRC cell lines harboring *KRAS* mutations (Fig. 5b). Finally, KY7749 successfully overcame the ineffectiveness of cetuximab and inhibited the migration of metastatic CRC LoVo cells with *KRAS* mutations (Fig. 5c). To determine the combined effect of KY7749 and cetuximab on CRC cell growth, the appropriate concentration of cetuximab (0.05 μg ml^−1^; almost 40% reduction in D-WT cell growth) was selected for combination treatment with KY7749 (Supplementary Figure S7). The combined effect of KY7749 and cetuximab was evident only in D-WT cells (Fig. 5d). Overall, KY7749 was shown inhibit the growth, transformation, and migration of *KRAS*-mutant cetuximab-insensitive CRC cells.

## Discussion

*RAS* gene mutations are a major cause of human cancer, and the resulting aberrant activation of downstream signaling pathways, such as the ERK and PI3 kinase-Akt pathways, contributes to malignant transformation and growth of various cancer types^29–31^. In particular, *KRAS* mutations play a major role in human cancers; notably, *KRAS* mutations have been observed in 40–50% of CRC patients^8, 32^. Efforts to develop anticancer drugs to control aberrant Ras activity have not been successful. However, the need to develop such drugs has been growing due to the failure of current drugs that target EGFR, which are frequently prescribed for the treatment of advanced metastatic CRC, are often insensitive to these drugs when the patients harboring *KRAS* mutations^33^. Therefore, the development of a drug that can be prescribed to patients harboring *KRAS* mutations is critical for the treatment of cancer patients.

Most previous approaches to control the activity of oncogenic mutant Ras have involved searches for small molecules that bind directly to Ras, farnesyltransferase inhibitors, and inducers of synthetic lethality^9–14^. As an alternative approach controlling Ras activity, on the basis of our studies illustrated the cross-talk between the Wnt/β-catenin and Ras-ERK pathways^23, 25, 34, 35^, we identified and characterized small molecules degrading Ras^20, 21^, especially oncogenic mutant K-Ras. These compounds and derivatives, KYA1797K and KY1022, inhibited the growth of CRC cells via degradation of both β-catenin and Ras^20, 21^. However, these compounds did not significantly degrade Ras in *CTNNB1*-mutant CRC cells.

Therefore, we screened 10,000 small molecules for their ability to degrade Ras, especially mutant K-Ras, independent of β-catenin by generating cells that can detect EGFP-K-Ras^G12V^ levels with high sensitivity in HCT-116 cells harboring *CTNNB1* mutations. We identified KY7749 as a candidate compound that most effectively degrades Ras in CRC cells with *CTNNB1* mutations. KY7749 suppressed growth, transformation, and metastatic migration of CRC cells harboring a *KRAS* mutation. Moreover, resistance to EGFR antibodies in *KRAS*-mutant CRC cells was successfully overcome with KY7749 treatment. This effect was achieved through degradation of the K-Ras protein. Combined growth inhibitory effects of KY7749 and cetuximab were only observed in WT *KRAS* CRC cells co-treated with KY7749 and low-doses of cetuximab. Absence of the combinatorial effect in *KRAS*-mutant cells is due to the effects of cetuximab, which inactivates Ras via reduction of GTP-bound Ras but does not influence mutant *RAS*. Next steps in the development of a clinically usable anticancer drug will include improvements to KY7749 efficacy and druggability through chemical synthesis and characterization of analogs.

Current approaches directed at the inhibition of CRC cell transformation, especially in those cells with *CTNNB1* mutations, can potentially be applied to the treatment of CRC and other types of cancers in patients displaying aberrant EGFR-Ras pathway activation caused by either mutations or the overexpression of EGFR and/or Ras. Lowering activities of Ras via degradations of their proteins could be an alternative approach for the development of anticancer drugs that can overcome the current limitations involving the undruggable nature of Ras in patients with *RAS* mutations.

## Electronic supplementary material

Supplementary Figures
