# Supplementary material for: Identification of Ras-degrading small molecules that inhibit the transformation of colorectal cancer cells independent of β-catenin signaling
Source: Exp Mol Med. 2018 Jun 6;50(6):1–10. doi: 10.1038/s12276-018-0102-5 (PMC5994827; doi:10.1038/s12276-018-0102-5)
Supplement: Supplementary file 1 — Supplementary Figures [file 12276_2018_102_MOESM1_ESM.doc]

**
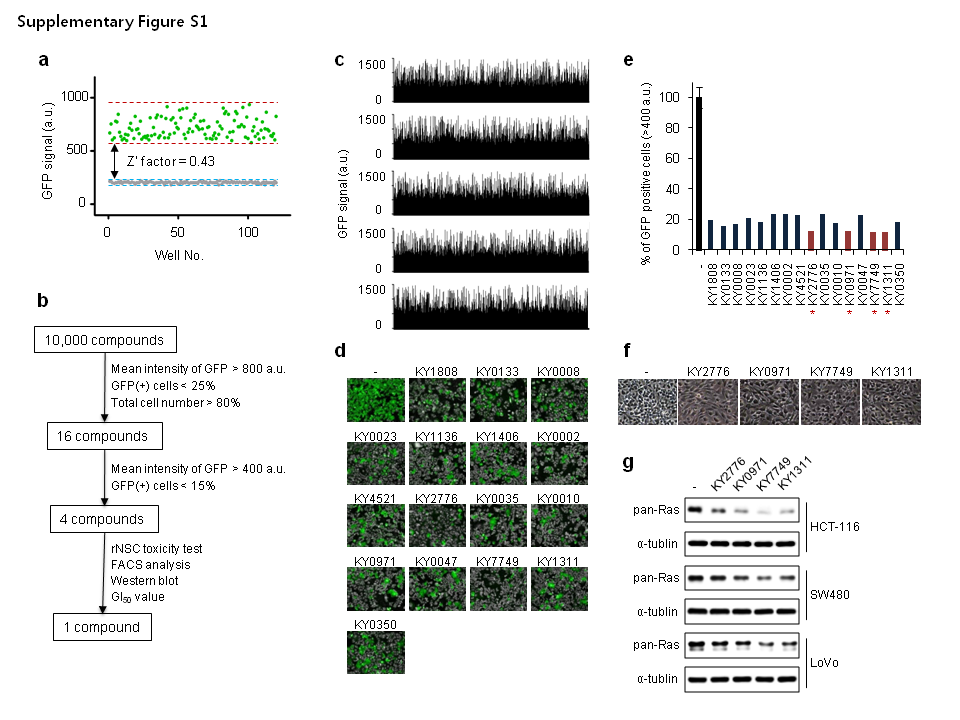
Supplementary Figure S1** Identification of Ras degrading small molecules by High-content screening (HCS) in *CTNNB1* mutant HCT-116 cells. (**a**) The fluorescence images of cells treated with four hit compounds selected by HCS. Z′ factor values of untreated HCT-116 EGFP-K-Ras^G12V^ (green dots) and HCT-116 parental cells (gray dots). HCT-116 parental cells were used as a positive control. (**b**) A flow scheme of the HCS used to identify KY7749 as a compound that decreased the EGFP-K-Ras^G12V^ signal in HCT-116 cells. In order to exclude compounds with significant toxic effects, compounds that reduced the total cell numbers more than 20% were filtered out. Compounds that reduced more than 75% of the total green fluorescent protein (GFP)-positive cells were selected as the initial hit compounds. For the detail analyses of the initial hit compounds, the reference point of GFP-positive cells was revised to over 400 arbitrary units (a.u.) mean intensity of GFP in the cytoplasm. Compounds that reduced more than 85% of the total GFP-positive cells were selected and further characterized to select the most effective compound. (**c**) Intensity of GFP signals for the cells treated with each of the 10,000 compounds. (**d**) The fluorescence images of 16 initial hit compounds. GFP-positive cells were over 800 a.u. mean intensity of GFP in the cytoplasm and were marked with a green color. (**e**) Quantification of GFP-positive cells treated with each of the 16 initial hit compounds. Four compounds that reduced GFP-positive cells more than 85% are indicated by red asterisks. (**f**) To measure cellular toxicity of the four hit compounds, undifferentiated rat neural stem cells (rNSCs) were treated with 10 μM of each compound for 48 h. (**g**) Immunoblot analyses of HCT-116, SW480, and LoVo cells treated with 10 μM each of the four hit compounds for 24 h. Whole cell lysates (WCLs) of each of the cells were immunoblotted with antibodies to indicated proteins (**g**).

**
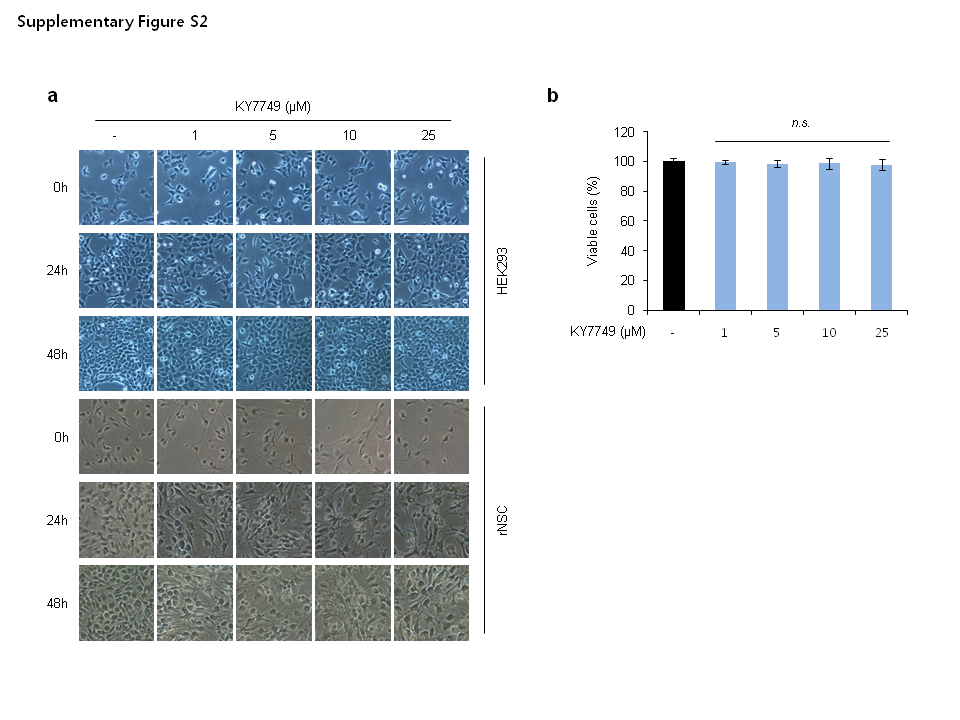
Supplementary Figure S2** The cytotoxic test of KY7749 in normal cells. HEK293 cells and rNSCs were treated with DMSO (control) or indicated doses (1 to 25 μM) of KY7749 for up to 48 h. (**a**) Morphology images were captured by bright field microscopy at 0, 24, and 48 h. (**b**) HEK293 cells treated with indicated doses of KY7749 for 48 h were subjected to cell viability assays (mean ± SD, n = 3). Viable cells were normalized to DMSO-treated controls.

**
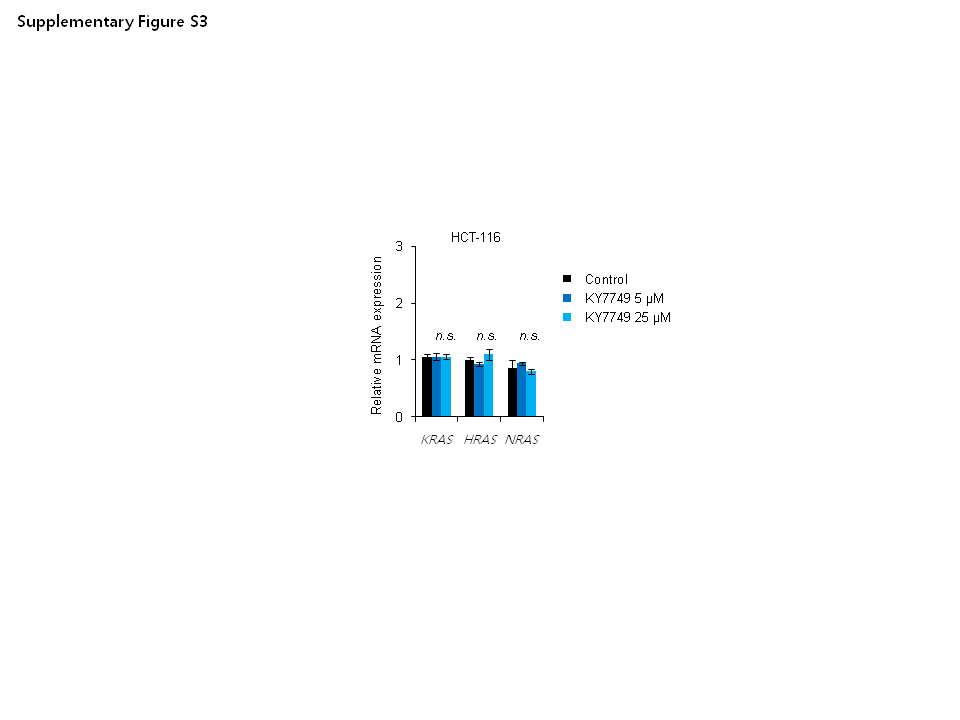
Supplementary Figure S3** The effects of KY7749 on mRNA expression of Ras isotypes. HCT-116 cells treated with DMSO (control), 5 μM, or 25 μM of KY7749 for 24 h were subjected to quantitative real-time polymerase chain reaction (qRT-PCR) analyses (mean ± SD, n = 3).

**
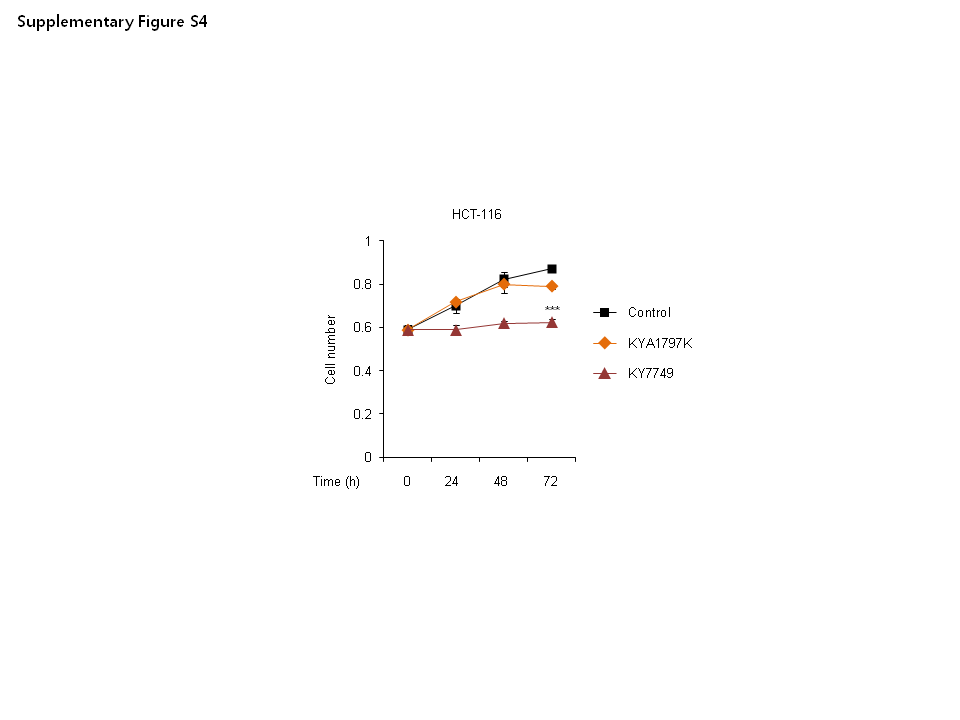
Supplementary Figure S4** The effects of KY7749 or KYA1797K on cell proliferation of *CTNNB1* mutant CRC cells. HCT-116 cells were treated with DMSO (control), 25 μM of KY7749, or KYA1797K for the indicated time periods. Relative cell proliferation was normalized to DMSO-treated controls (mean ± SD, n = 3).

**
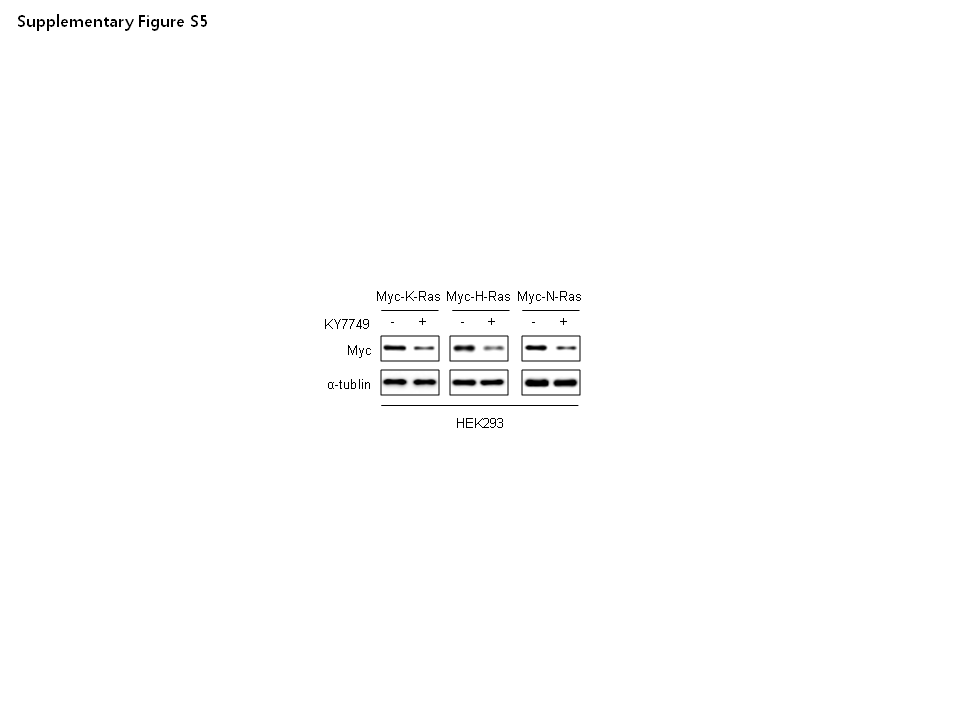
Supplementary Figure S5** The effects of KY7749 on degradation of Ras isotypes. HEK293 cells were overexpressed with Myc-K-Ras, Myc-H-Ras, or Myc-N-Ras, and treated with 25 μM of KY7749 for 24 h. WCLs were immunoblotted with each antibody for the indicated proteins.

**
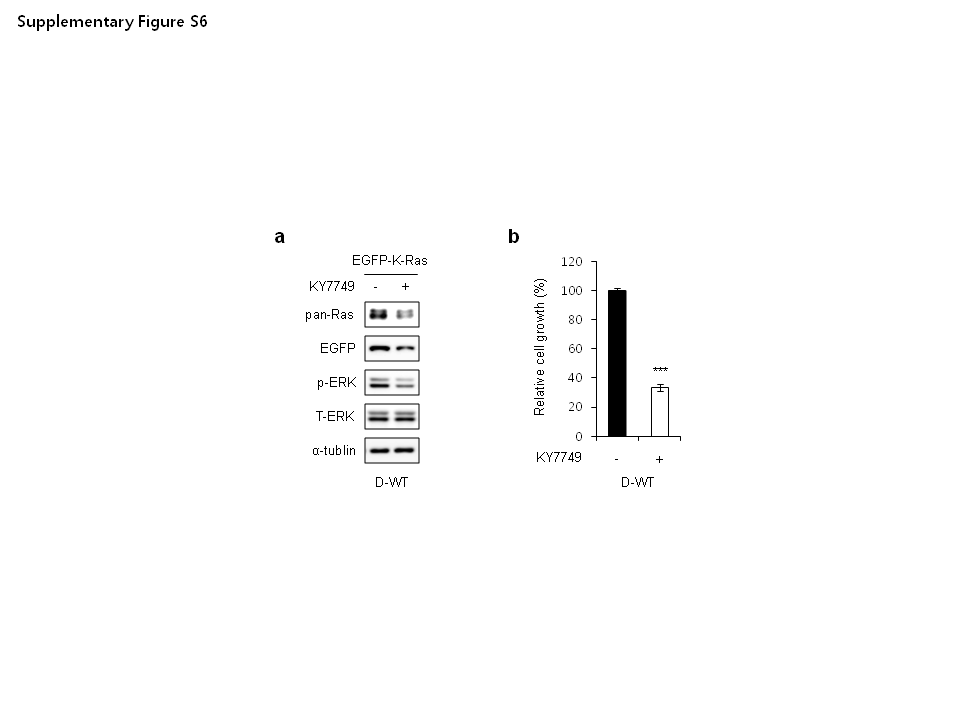
Supplementary Figure S6** The effects of KY7749 on WT Ras degradation and proliferation in CRC cells harboring WT *RAS*. (**a**) D-WT cells were overexpressed with EGFP-K-RAS, and treated with 25 μM of KY7749 for 24 h. WCLs were immunoblotted with each antibody for the indicated proteins. (**b**) D-WT cells were treated with DMSO (control) or 25 μM of KY7749 for 96 h. Relative cell proliferation was normalized to DMSO-treated controls (mean ± SD, n = 3).


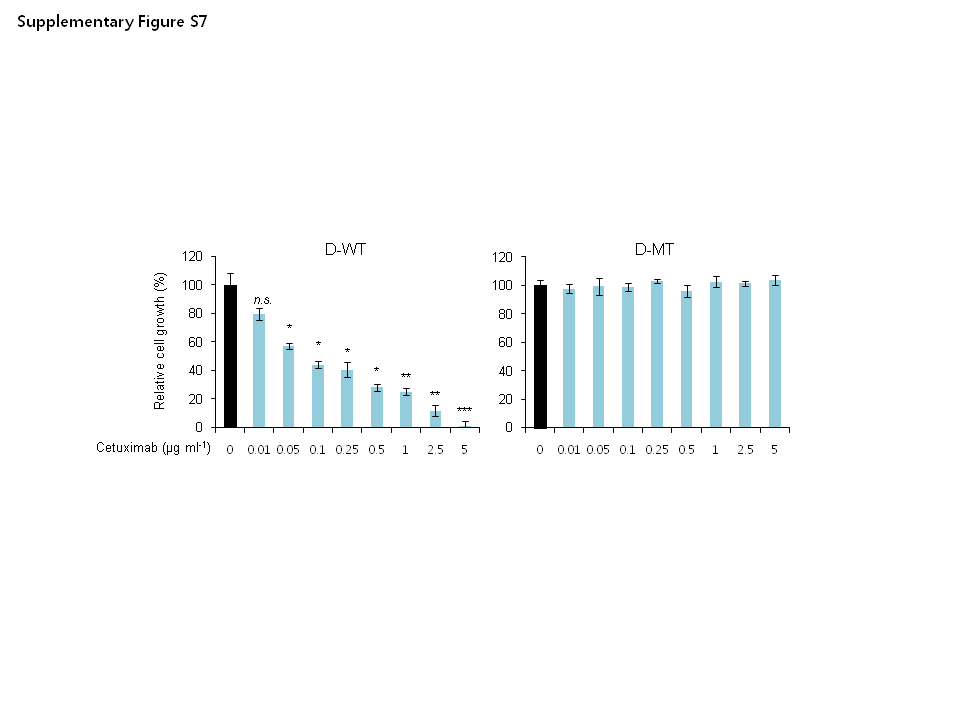
**Supplementary Figure S7** The effects of cetuximab on growth of CRC cells. D-WT or D-MT cells were treated with indicated concentration (0.01 to 5 μg ml^-1^) of cetuximab for 96 h. Relative cell proliferation was normalized to DMSO-treated controls (mean ± SD, n = 3).
